# Quality of Life in relation to Vitamin-D levels in asymptomatic young health care professionals in a tertiary care hospital: Quality of Life and Vitamin-D (QoLD) study

**DOI:** 10.12669/pjms.42.3.11603

**Published:** 2026-03

**Authors:** Azizul Hasan Aamir, Shaista Kanwal, Azmat Ali Khan

**Affiliations:** 1 Azizul Hasan Aamir, MBBS(Pakistan), MRCP(UK), FRCP(UK), FACE(US), CCST (Diabetes/Endocrinology and Int. Medicine). Department of Diabetes, Endocrinology and Metabolic Diseases, MTI Hayatabad Medical Complex, Peshawar, Pakistan; 2 Shaista Kanwal, MBBS(Pakistan), MRCP (UK), FCPS (Medicine), FCPS (Endocrinology), Department of Diabetes, Endocrinology and Metabolic Diseases, MTI Hayatabad Medical Complex, Peshawar, Pakistan; 3 Azmat Ali Khan, MBBS(Pakistan), FCPS (Medicine), Department of Diabetes, Endocrinology and Metabolic Diseases, MTI Hayatabad Medical Complex, Peshawar, Pakistan

**Keywords:** Quality of life, Vitamin-D, SF 36, Health care professionals, 25-hydroxyVitamin-D, Health-related quality of life, Pakistan

## Abstract

**Objectives:**

We have assessed the Vitamin-D status and its association with health-related quality of life in healthy young health care professionals in a tertiary care hospital in Pakistan.

**Methodology:**

This cross-sectional study was performed in the Department of Diabetes, Endocrinology and Metabolic Diseases, Hayatabad Medical Complex, Peshawar, Pakistan. Three hundred and nineteen healthy young health care professionals (HCPs) aged 20-40 years of age were enrolled in July 2023 using non-probability convenient sampling method. Vitamin-D status was categorized as deficient (<20 ng/ml), insufficient (21-29 ng/ml), or sufficient (≥30 ng/ml) per Endocrine Society guidelines. The Medical Outcomes Study 36-Item Short Form Health Survey measured health related quality of life (HRQoL). ANOVA compared continuous variables, while Chi-square tested categorical variables. A p value < 0.05 indicated statistical significance in the two-sided analysis.

**Results:**

From July 1st to July 31st, 2023, 319 individuals were recruited, with a mean age of 28.2 ± 4.96 years, with 230 (72.1%) males and 89 (27.9%) females. The mean serum 25-hydroxyVitamin-D concentration was 24.9 ± 9.55 ng/ml with 82.1% being either deficient or insufficient. The mean physical component score (PCS), mental component score (MCS) and total short form-36 (SF-36) score were 81.2 ± 11.4, 77.9 ± 12.9 and 79.6 ± 11.1 respectively. Vitamin-D did not affect total SF-36 score (p=0.09). Only role limitation due to emotional issues (p=0.04), energy/fatigue (p=0.04), and emotional wellness (p=0.03) showed significant differences, with lower scores in the deficient group.

**Conclusion:**

Most healthy young HCPs were Vitamin-D insufficient, whilst 95% had a mean SF-36 score >50. However, there was no relationship between Vitamin-D status and the quality of life.

## INTRODUCTION

The global Vitamin-D insufficiency rate is 24%-49%.^1^ The level of Vitamin-D in the body is assessed by measuring serum 25-hydroxyVitamin-D [25(OH)D], which is the main circulating metabolite and the best indicators of Vitamin-D stores. It is derived from dietary cholecalciferol (Vitamin-D3) and skin synthesis.^2^ Pakistan is a ’hot zone’ for Vitamin-D insufficiency, with 61-65% of the population having 25 (OH)D levels below 10 ng/ml, according to a systematic analysis of low- and middle-income countries (LMICs).^3^ Despite ample sunlight, this high prevalence is attributed to inadequate dietary fortification, indoor lifestyle, occupational factors reducing exposure to sun, and cultural clothing practices covering skin.^4^ Vitamin-D supplements are believed to improve musculoskeletal health, however data is mixed.^5^ Vitamin-D insufficiency is linked to cardiovascular illness, musculoskeletal disorders, cancers, autoimmune disease, and type-2 diabetes mellitus, underscoring its importance in health.^6^

Recent evidence questions Vitamin-D benefits. The Vitamin-D and OmegA-3 TriaL (VITAL) and Vitamin-D3-Omega3-Home Exercise-Healthy Aging and Longevity (DO-Health) trials found no Vitamin-D benefit for musculoskeletal health.^7^ In a systematic review and meta-analysis of 80 randomized controlled trials, Vitamin-D did not reduce cardiovascular morbidity and death.^7^ Similarly, a systematic review found no convincing evidence that Vitamin-D improves mental health and quality of life.^8^ In view of these findings, the meta-analysis by Bolland et al has suggested for the reformulation of guidelines on Vitamin-D supplementation in relation to chronic disease.^9^

Despite abundant sunlight, most young individuals in Pakistan and India are Vitamin-D deficient.^1^,^10^ Healthcare professionals are more prone to Vitamin-D deficiency due to limited exposure to sun, indoor working hours, and shift work.^4^ However, the association between Vitamin-D status and health-related quality of life in this population remains unclear. Moreover, gender differences in Vitamin-D status are well documented, with females typically having lower levels than males due to clothing preferences, reduced sun exposure, and physiological factors.^4^

The main objective of this study was to establish the frequency of Vitamin-D insufficiency and deficiency in healthy young health care professionals (HCPs) and to determine its association with quality of life. A secondary objective was to assess the association with parathyroid hormone (PTH), phosphorus, calcium, and alkaline phosphatase levels.

## METHODOLOGY

The study was conducted from July 1 to July 31, 2023. The study was conducted exclusively in July to capture participants during the period of maximum sunlight exposure in Pakistan, thereby representing the best-case scenario for Vitamin-D synthesis through sun exposure. After informed consent, participants joined. Given that the prevalence of Vitamin-D deficiency in Pakistan is 71.5%^11^, with a confidence interval of 95% and margin of error of 5%, the sample size was estimated to be 314, using the open Epi sample size calculator.^12^

### Ethical Approval:

The hospital’s ethical review committee approved this cross-sectional study in July 2023 at Medical Teaching Institute (MTI)-Hayatabad Medical Complex, Peshawar (Approval No. 857 Dated October 14, 2022).

Three hundred and nineteen healthy young HCPs aged 20-40 were enlisted via non-probability convenience sampling from various departments including medical wards, surgical units, emergency department, outpatient clinics, and administrative offices. The sample comprised 153 doctors (48%), 157 nursing and paramedical staff (49.2%), and 9 medical students (2.8%), with 230 males (72.1%) and 89 females (27.9%). Both males and females’ participants fulfilling the inclusion criteria were enrolled.

### Inclusion Criteria:

No specific gender selection criteria were applied. Participants were considered healthy based on:

- self-reported good status of health,
- no current acute illness,
- absence of chronic medical conditions, and
- self-reported good health status. Asymptomatic status was determined through a screening questionnaire assessing absence of:
- chronic fatigue or generalized weakness,
- musculoskeletal pain or myalgia,
- bone pain or joint aches,
- mood disturbances or depression,
- frequent headaches, and
- any acute illness in the preceding month. Only participants reporting no such symptoms were included.

Excluded were HCPs with metabolic bone disorders, those who had taken supplemental Vitamin-D within the preceding three months, pregnant or lactating women, celiac disease, inflammatory bowel disease, or taking Vitamin-D-affecting medications like antiepileptics, dexamethasone, spironolactone, nifedipine, rifampin, antithyroid drugs, and clotrimazole.

Participants’ demographics, lifestyle (sun exposure, sun block, and exercise), and food (milk, eggs, fish, and beverages) information were collected using a predesigned questionnaire. Sun exposure was quantified by asking participants: ’On average, how many days per week is your skin exposed to direct sunlight for at least 15 minutes?’ Dietary intake was assessed for daily milk consumption (in milliliters), fish intake (servings per week), and egg consumption (number per week). The Medical Outcomes Study 36-Item Short Form Health Survey (SF-36) version 2.0 examined HRQoL using 36 questions covering eight health domains: physical function (PF), role limitation due to physical health (RP), body pain (BP), general health perception (GH), vitality/energy (VT), social functioning (SF), role limitation due to emotional problems (RE), and emotional wellbeing (EH). Domain scores range from 0 to 100, with greater scores meaning better health. Two summary scores are calculated: Physical Component Score (PCS) derived from PF, RP, BP, and GH domains, and Mental Component Score (MCS) derived from VT, SF, RE, and EH domains. Both component scores are standardized to a mean of 50. Scores >50 indicate good quality of life, while scores <50 indicate poor quality of life.^13^

The following laboratory parameters were measured: 25(OH) Vitamin-D, calcium (Ca), phosphorus, alkaline phosphatase (AP), hemoglobin (Hb), and intact parathyroid hormone (PTH). Serum 25-(OH) D levels were measured using standard immunoassay technique at the hospital laboratory. Endocrine society guidelines were adopted to define Vitamin-D deficiency [25(OH) D < 20 ng/ml (50 nmol/liter)], insufficiency [25(OH)D of 21-29 ng/ml (52.5-72.5 nmol/liter)] and sufficiency (>30 ng/ml (75 nmol/liter).^14^ These cutoffs remain the current standard in clinical practice.^15^ Normal reference ranges for other laboratory parameters were: intact parathyroid hormone (PTH) 10-65 pg/ml, serum calcium 8.5-10.5 mg/dl, serum phosphorus 2.5-4.5 mg/dl, alkaline phosphatase 30-120 U/L, and hemoglobin 12-16 g/dl for females and 13-17 g/dl for males.

### Data Analysis:

Data was analyzed using SPSS (20.0). Continuous variables were represented by means with SD or 95% confidence intervals, whereas categorical variables were frequencies. ANOVA compared continuous variables, while Chi-square tested categorical variables. Vitamin-D levels, SF-36 scores, serum calcium, phosphorus, alkaline phosphatase, PTH, and hemoglobin were examined using Spearman correlation coefficients. A p value < 0.05 indicated statistical significance in the two-sided analysis.

## RESULTS

From July 1, 2023 to July 31, 2023, 319 HCPs participated in the study of whom, 153 (48%) were doctors, 157 (49.2%) were nursing and paramedical staff and 9 (2.8%) were medical students. Of the 319 participants, 230 (72.1%) were males and 89 (27.9%) were females. Mean age was 28.2 ± 4.9 years, while the mean BMI was 23.9 ± 4.1 kg/m^2^. Of the participants, 226 (70.8%) had never used Vitamin-D supplementation, 28 (8.8%) had used Vitamin-D 3 to 6 months prior to enrollment (beyond the three-month exclusion period), and 65 (20.4%) had used Vitamin-D more than six months prior. The mean 25(OH)D was 24.9 ± 9.55 ng/ml, range 8.9 ng/ml to 72.7 ng/ml with 262 (82.1%) having a Vitamin-D <30 ng/ml, whilst 100 (31.3%) had Vitamin-D <20 ng/ml. The mean Vitamin-D in males (26.6 ± 9.6 ng/ml) was significantly higher compared to females (20.6 ± 7.9 ng/ml) (p<0.001). Approximately 57.3% of the females and 21.3% of the males had deficient Vitamin-D levels and 56.5% of females and 36% of males were insufficient.

The mean PTH was 29.2 ± 10.6 pg/ml, ranging from 2.7 to 74 pg/ml. PTH levels did not differ significantly between Vitamin-D deficient (30.4 ± 10.5 pg/ml), insufficient (29.1 ± 10.5 pg/ml), and sufficient (27.1 ± 10.7 pg/ml) groups. A substantial link exists between Vitamin-D levels and PTH (r= -0.131, p= 0.02). The mean calcium level was considerably lower (9.6 ± 0.4 mg/dl) in the deficient group compared to the sufficient (p<0.001) and insufficient (p=0.001) groups. The mean phosphorus level was 3.3 ± 0.56 mg/dl, with no variations across groups (p=0.21). Mean alkaline phosphatase level was 87.8 ± 23.9 mg/dl, no difference between groups (p=0.2). The mean PCS, MCS and total SF 36 score were 81.2 ± 11.4, 77.9 ± 12.9 and 79.6 ± 11.1, respectively (Table-I). Three SF-36 domains differed significantly across Vitamin-D categories: role limitation due to emotional problems (p=0.04), energy/fatigue (p=0.04), and emotional wellbeing (p=0.03). The deficient group had lower scores in these domains compared to insufficient and sufficient groups (Table-I), though differences were modest. There was a significant difference (p < 0.001) between men and women in the number of people who had deficient, sufficient, and insufficient Vitamin-D. (Table-II).

**Table-I:** Differences in levels of Vitamin-D with quality of life.

SF 36 Domain	Vitamin-D categories*	Number of participants	Mean Quality of life domain score	Standard deviation	95% confidence interval for mean		p-Value
					Lower bound	Upper bound	
1.Physical functioning	0 to 20	100	86.5	15.7	83.3	89.6	0.42
	21 to 29	162	88.7	16.5	86.2	91.3	
	30 to 100	57	89.4	13.9	85.7	93.1	
	Total	319	88.1	15.8	86.4	89.9	
2.Role limitation due to Physical health	0 to 20	100	86.2	19.2	82.3	89.9	0.19
	21 to 29	162	90.1	16.8	87.5	92.7	
	30 to 100	57	90.1	19.0	85.04	95.1	
	Total	319	88.9	18.0	86.9	90.8	
3.Role limitation due to emotional problems	0 to 20	100	80.4	25.9	75.3	85.6	0.04
	21 to 29	162	87.8	20.8	84.5	90.9	
	30 to 100	57	86.0	21.8	80.2	91.8	
	Total	319	85.2	22.9	82.6	87.7	
4.Energy/Fatigue	0 to 20	100	70.9	13.8	68.1	73.6	0.04
	21 t0 29	162	74.9	14.5	72.7	77.2	
	30 to 100	57	70.6	16.1	66.3	74.9	
	Total	319	72.9	14.7	71.3	74.5	
5.Emotional wellbeing	0 to 20	100	73.8	12.7	71.2	76.3	0.03
	21 to 29	162	76.9	11.9	75.03	78.7	
	30 to 100	57	72.5	13.9	68.8	76.1	
	Total	319	75.1	12.6	73.7	76.5	
6.Social Functioning	0 to 20	100	78.8	15.9	75.6	82.0	0.91
	21 to 29	162	78.9	14.9	76.6	81.3	
	30 to 100	57	77.8	18.8	72.9	82.8	
	Total	319	78.7	15.9	76.9	80.5	
7.Pain	0 to 20	100	75.8	17.99	72.2	79.3	0.30
	21 to 29	162	78.8	16.4	76.2	81.3	
	30 to 100	57	76.04	16.6	71.7	80.4	
	Total	319	77.4	16.9	77.5	79.2	
8.General Health	0 to 20	100	70.7	13.5	68.05	73.4	0.75
	21 to 29	162	70.8	13.2	68.7	72.8	
	30 to 100	57	69.3	14.97	65.3	73.2	
	Total	319	70.5	13.6	69.0	72.0	
9.Physical Component Score	0 to 20	100	79.8	10.7	77.7	81.9	0.27
	21 to 29	162	82.1	11.5	80.3	83.9	
	30 to 100	57	81.2	12.1	78.0	84.4	
	Total	319	81.2	11.4	79.9	82.5	
10.Mental Component Score	0 to 20	100	75.97	13.5	73.3	78.6	0.06
	21 to 29	162	79.6	12.01	77.8	81.5	
	30 to 100	57	76.7	14.0	73.03	80.5	
	Total	319	77.9	12.9	76.5	79.4	
Total SF 36 Score	0 to 20	100	77.9	10.9	75.6	80.1	0.09
	21 t0 29	162	80.9	10.8	79.2	82.6	
	30 to 100	57	78.97	12.3	75.7	82.3	
	Total	319	79.6	11.1	78.4	80.8	

* Vitamin-D categories: Deficient (0-20 ng/ml), Insufficient (21-29 ng/ml), Sufficient (30-100 ng/ml) based on Endocrine Society Clinical Practice Guidelines. P-values calculated using one-way ANOVA.

**Table-II:** The relationship between Vitamin-D levels and study variables.

Study Variables	Vitamin-D Categories*					p-Value
		Vitamin-D Deficient (0 -20 ng/ml) n= 100 (%)	Vitamin-D Insufficient (21 - 29 ng/ml) n= 162 (%)	Vitamin-D Sufficient (30 -100 ng/ml) n= 57 (%)	Total n= 319 (%)	
Gender	Male	49 (49)	130 (80.2)	51 (89.5)	230 (72.1)	< 0.001
	Female	51 (51)	32 (19.7)	6 (10.5)	89 (27.9)	
BMI	< 18.5	9 (9)	6 (3.7)	6 (10.5)	21 (6.6)	0.5
	18.5-22.9	33 (33)	52 (32.1)	19 (33.3)	104 (32.6)	
	23-24.9	24 (24)	44 (27.2)	16 (28.1)	84 (26.3)	
	≥ 25	34 (34)	60 (37.04)	16 (28.1)	110 (34.5)	
Exposure to sunlight (days per week)	No exposure	3 (3)	4 (2.5)	4 (7)	11 (3.4)	0.15
	Up to 3 days per week	35 (35)	42 (25.9)	13 (22.8)	90 (28.2)	
	4 to 6 days per week	35 (35)	65 (40.1)	16 (28.1)	116 (36.4)	
	Daily exposure	27 (27)	51 (15.9)	24 (42.1)	102 (31.9)	
	Skin not exposed	46 (46)	81 (50)	35 (61.4)	162 (50.8)	
Skin exposure	Skin exposed	54 (54)	81 (50)	22 (38.6)	157 (49.2)	0.17
	No milk intake	59 (59)	101 (62.3)	25 (43.9)	185 (57.9)	
Milk intake (per day)	Less than 100 ml	26 (26)	38 (23.5)	24 (42.1)	88 (27.6)	0.23
	100 to 199 ml	13 (13)	19 (11.7)	7 (12.3)	39 (12.2)	
	More than 200 ml	2 (2)	4 (2.5)	1 (1.7)	7 (2.2)	
	None	62 (62)	87 (53.7)	29 (50.9)	178 (55.8)	
Fish intake (per week)	Up to 2 times	37 (37)	71 (43.8)	28(49.1)	136 (42.6)	0.36
	3 to 6 times	1 (1)	4 (2.5)	0 (0)	5 (1.6)	
	No Exercise	30 (30)	35 (21.6)	13 (22.8)	78 (2.4)	
Exercise (per week)	Up to 3 days	18 (18)	22 (13.6)	6 (10.5)	46 (1.4)	0.35
	4 to 6 days	32 (32)	56 (34.6)	19 (33.3)	107 (33.5)	
	Daily	20 (20)	49 (30.2)	19 (33.3)	88 (27.6)	

* Vitamin-D categories as defined in Table I. P-values calculated using Chi-square test for categorical variables.

## DISCUSSION

This study defined Vitamin-D deficiency as serum 25(OH)D levels below 20 ng/ml, insufficiency as levels between 21 and 29 ng/ml, and sufficiency as levels above 30 ng/ml, according to Endocrine Society guidelines.^14^ In our study, 82.1% of healthy young HCPs had Vitamin-D levels below 30 ng/ml (31.3% deficient <20 ng/ml, 50.8% insufficient 21-29 ng/ml), with only 17.9% having sufficient levels. These findings are comparable to other studies. A Pakistani survey of young medical students found 79.4% Vitamin-D deficiency.^16^ Another study by Nadeem et al. found that 89.1% of asymptomatic healthy medical students in Karachi had Vitamin-D deficiency, while just 3.6% had adequate Vitamin-D^17^, compared to 17.9% in our study. A review study found that 64.6% of healthy individuals had Vitamin-D levels below 30 ng/ml.^18^ A study of southern Indian medical students revealed 89% had deficient Vitamin-D.^19^ Thus, most healthy young medical students and doctors are Vitamin-D deficient, especially in Pakistan and India, which get plenty of sun. Our investigation was conducted in July, amid long summer days and considerable sun exposure. Despite Pakistan’s ample sunlight all year long, the high prevalence of Vitamin-D insufficiency in our HCPs can be attributed to several occupational and lifestyle factors: prolonged indoor working hours in hospital settings (often 8-12 hour shifts), shift work patterns that reduce daytime outdoor exposure, cultural and occupational dress codes covering most skin areas, regular use of sunscreen for skin protection, and limited time for outdoor activities due to demanding work schedules. These factors collectively override the theoretical advantage of geographic sun exposure.^4^,^10^

Our study demonstrated that Vitamin-D deficient, insufficient, and sufficient groups did not differ in QoL, except for role limitation due to emotional issues, energy/fatigue, and emotional wellness. Various studies have assessed the association of Vitamin-D with HRQoL. A systematic review revealed no compelling evidence that Vitamin-D enhances mental health and quality of life.^7^ Vitamin-D levels did not significantly affect HRQoL except for depression and anxiety in a Korean study.^20^ Another Indonesian medical student study found that Vitamin-D intake did not affect quality of life in 17-35-year-olds.^21^ These results are comparable to our research, even though the former utilized EuroQol-5 to assess HRQoL in the general population and the latter employed WHOQOL-BREF.

Our results are consistent with the increasing evidence that challenges the causal link between Vitamin-D levels and subjective well-being indicators..^7^,^8^ Recent high-quality trials and meta-analyses increasingly suggest that earlier observational associations may reflect residual confounding rather than true causation, particularly in asymptomatic populations without overt deficiency-related symptoms.^22^,^23^ In a systematic analysis by Hoffman et al., short-term Vitamin-D supplementation improved HRQoL, but long-term supplementation did not.^23^ This systematic review acknowledged study design variations, HRQoL instrument discrepancies, and Vitamin-D type and dose. The lack of correlation between Vitamin-D levels and quality of life in our study may reflect a ceiling effect rather than true independence. With 98.4% of participants maintaining SF-36 scores ≥50 and the population being young, healthy, and asymptomatic by design, there was minimal variation in quality-of-life scores to detect meaningful associations. This ceiling effect is a recognized limitation when studying HRQoL in healthy populations.^23^

We performed ROC curve analysis to assess whether Vitamin-D levels could predict quality of life impairment. The outcome variable was defined as SF-36 total score <50 (poor quality of life), while Vitamin-D level was the predictor variable. Among 319 participants, only 5 (1.6%) had SF-36 scores <50, while 314 (98.4%) had scores ≥50, reflecting the overall good health status of this young, health population. The ROC analysis identified an optimal Vitamin-D cutoff of 23.45 ng/ml with sensitivity of 20% and specificity of 48.7%. The area under curve was 0.58 (95% CI: 0.316-0.848, p=0.528), indicating poor discriminative ability. The 2x2 contingency table showed: true positives=1, false positives=161, true negatives=153, false negatives=4. These findings confirm that Vitamin-D level is not a useful predictor of quality-of-life status in this population, as the AUC of 0.58 is only marginally better than chance (0.50). The very low sensitivity (20%) further indicates poor detection of quality-of-life impairment based on Vitamin-D levels alone. A study of healthy school children found that the cutoff point for blood 25(OH)D, which led to an increase in serum PTH, was 14.3ng/ml (area under the curve 0.622, CI 95%, sensitivity 78%, specificity 40%, p < 0.0001).^24^

**Fig.1:**
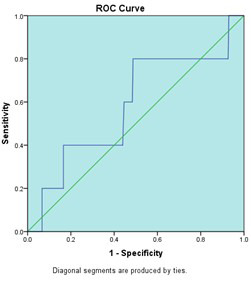
Receiver Operating Characteristic (ROC) curve for Vitamin-D level as predictor of quality of life impairment (SF-36 total score <50). The area under curve (AUC) of 0.58 (95% CI: 0.316-0.848, p=0.528) indicates poor discriminative ability, only marginally better than chance (AUC=0.50). Optimal cutoff: 23.45 ng/ml; Sensitivity: 20%; Specificity: 48.7%.

We found a substantial Vitamin-D-PTH association, similar with recent studies.^25^,^26^ Despite 82.1% having Vitamin-D < 30 ng/ml, none developed secondary hyperparathyroidism, possibly due to adequate calcium. PTH began to rise when Vitamin-D went below 15.8 ng/ml^21^ in one study and 10 ng/ml in another study in Saudi Arabia.^25^ While iPTH measurement is expensive and not recommended for routine screening of Vitamin-D deficiency in healthy individuals, it was included in this research protocol to provide pathophysiological insights into the Vitamin-D-PTH relationship in our population. Our approach helped us understand the metabolic effects of Vitamin-D insufficiency and establish the threshold at which PTH rises, which has important scientific implications but is not practical for routine clinical screening.

This research makes significant contributions to literature. First, it is one of the first studies to use a validated HRQoL instrument to look at the link between Vitamin-D levels and health-related quality of life in South Asian HCPs. Second, it offers current data on the significant prevalence of Vitamin-D insufficiency (82.1%) among young and healthy individuals, despite the geographic advantage of ample sunlight, underscoring the primary influence of occupational and lifestyle factors over environmental factors. Third, the absence of a correlation between Vitamin-D status and quality of life in healthy young adults undermines the justification for universal screening predicated solely on well-being outcomes.

This study has a lot of clinical significance. Even though 82.1% of people had Vitamin-D levels below 30 ng/ml by international standards, 98.4% of them had a good quality of life. This suggests that cutoff values from the West may not work for South Asian populations. Our results suggest that individuals from Pakistan may be functionally adequate at reduced Vitamin-D levels, potentially attributable to genetic or physiological adaptations. Utilizing international thresholds may result in the overdiagnosis of ’deficiency’ in populations that are, in fact, adequate at lower levels, leading to unnecessary screening and unjustified supplementation. Instead, universal application of Western standards, South Asian populations should have their own reference ranges for Vitamin-D status.

The study’s strengths comprise a relatively substantial sample size (n=319), the utilization of a validated SF-36 instrument, a thorough evaluation of various quality of life domains, the incorporation of PTH measurement to assess physiological effects, and a concentration on a previously under-researched occupational group.

### Limitations:

This study possesses several limitations. First, the use of non-probability convenience sampling of young, healthy, educated HCPs significantly restricts the external validity and generalizability of our findings. The results are not applicable to the general population, older adults, individuals with comorbidities, or those from lower socioeconomic backgrounds. Second, we only enrolled young HCPs (aged 20 to 40) who already had a better quality of life than the general population, which may explain the lack of association between Vitamin-D status and quality of life. Third, all participants were well-educated professionals from moderate to high income levels, further limiting generalizability. Fourth, serum calcium levels were not adjusted for albumin, potentially compromising the precision of calcium status assessment. Nonetheless, considering that all participants were young (20-40 years) and healthy, the probability of substantial hypoalbuminemia or protein abnormalities influencing calcium measurements was negligible. Fifth, the cross-sectional design prevents the determination of causal relationships between Vitamin-D status and quality of life. Sixth, we did not conduct multivariate regression analysis to adjust for potential confounders such as BMI, gender, dietary intake, sun exposure patterns, and sunscreen use.

The use of only bivariate analyses (ANOVA and Chi-square tests) may have obscured subtle associations between Vitamin-D and health-related quality of life that could emerge after controlling these variables. Seventh, self-reported dietary intake and sun exposure data are susceptible to recall bias and may not accurately represent actual exposure patterns. Finally, non-probability convenience sampling may have caused selection bias, as participants who volunteered may differ from non-participants in unmeasured ways. Based on the results of this study, large scale longitudinal studies with multiple centers and middle-aged and elderly people from the general population from different socioeconomic classes should be conducted to assess Vitamin-D status across all age groups and population categories.

Future research should also include randomized controlled trials of Vitamin-D supplementation in symptomatic individuals, examination of non-QoL health outcomes, and establishment of population-specific reference ranges for South Asian populations. This study demonstrates that 82.1% of healthy young HCPs have Vitamin-D insufficiency (<30 ng/ml), yet 98.4% maintain good quality of life (SF-36 ≥50). Vitamin-D deficiency was not associated with health-related quality of life, despite its high frequency. From a public health standpoint, our findings pose significant concerns regarding the routine Vitamin-D screening in asymptomatic young adults. Universal screening and supplementation initiatives aimed at this demographic, based solely on quality-of-life outcomes, may lack cost-effectiveness or clinical justification. These findings suggest that Vitamin-D screening in healthy young adults may not be justified based on quality-of-life outcomes alone, though screening for other clinical indications should be considered individually. Moreover, Vitamin-D deficiency had a modest impact on quality of life. Based on these findings, our population may have different Vitamin-D deficiency cutoffs.

### Author’s Contribution:

**AHA:** Conceived, designed, literature review, statistical analysis, interpretation of data, and critically revised the manuscript.

**SK AAK:** Did literature review, data collection, statistical analysis, interpreted the data and helped in drafting the manuscript.

AHA and SK take joint responsibility for the overall integrity and accuracy of the work

All authors have read and approved the final manuscript.
